# Humanized Mouse Models for the Advancement of Innate Lymphoid Cell-Based Cancer Immunotherapies

**DOI:** 10.3389/fimmu.2021.648580

**Published:** 2021-04-22

**Authors:** Nina B. Horowitz, Imran Mohammad, Uriel Y. Moreno-Nieves, Ievgen Koliesnik, Quan Tran, John B. Sunwoo

**Affiliations:** ^1^ Department of Otolaryngology-Head and Neck Surgery, Stanford Cancer Institute and Institute for Stem Cell Biology and Regenerative Medicine, Stanford University School of Medicine, Stanford, CA, United States; ^2^ Department of Bioengineering, Stanford University School of Medicine and School of Engineering, Stanford, CA, United States

**Keywords:** humanized mice, innate lymphocyte cells, natural killer cell, cancer immunotherapy, oncoimmunology, PDX models

## Abstract

Innate lymphoid cells (ILCs) are a branch of the immune system that consists of diverse circulating and tissue-resident cells, which carry out functions including homeostasis and antitumor immunity. The development and behavior of human natural killer (NK) cells and other ILCs in the context of cancer is still incompletely understood. Since NK cells and Group 1 and 2 ILCs are known to be important for mediating antitumor immune responses, a clearer understanding of these processes is critical for improving cancer treatments and understanding tumor immunology as a whole. Unfortunately, there are some major differences in ILC differentiation and effector function pathways between humans and mice. To this end, mice bearing patient-derived xenografts or human cell line-derived tumors alongside human genes or human immune cells represent an excellent tool for studying these pathways *in vivo*. Recent advancements in humanized mice enable unparalleled insights into complex tumor-ILC interactions. In this review, we discuss ILC behavior in the context of cancer, the humanized mouse models that are most commonly employed in cancer research and their optimization for studying ILCs, current approaches to manipulating human ILCs for antitumor activity, and the relative utility of various mouse models for the development and assessment of these ILC-related immunotherapies.

## Introduction

Natural killer (NK) cells are lymphocytes of the innate immune system capable of killing cancerous or precancerous cells as well as virally infected cells. NK cell functions are tightly regulated by the balance of signals from activating receptors and inhibitory receptors (1, 2). It was first observed in the 1970s that these “naturally occurring killer lymphocytes” can kill tumor cell lines without the need for prior sensitization (3, 4). Later studies in mice showed an increase in tumor growth and metastasis after NK cells were depleted genetically or therapeutically, supporting the crucial role of NK cells in cancer immunosurveillance (5, 6).

NK cells belong to a family of immune cells called innate lymphoid cells (ILCs) (7). The ILC family is a heterogeneous class of immune cells that are increasingly studied for their emerging roles in cancer. ILC family members have differential effects on tumors; some ILC subgroups inhibit tumor growth while others promote it (8). Numerous studies have contributed to the understanding of mouse ILC biology (9). It is important to note, however, that there are species-specific markers and potential developmental differences between mouse and human ILC subsets (10–12).

Human ILCs, especially NK cells, have been leveraged to treat cancer through a variety of modalities including antibody-based therapies, cell-based therapies and bioengineered immunomodulatory therapies. While some of these therapies have shown remarkable success, especially with hematopoietic cancers, many are limited in their efficacy against solid tumors (13–15). This highlights the need for solid tumor mouse models that allow for more accurate studies of human ILCs and their related therapies.

Many murine models have been developed to study cancer development and treatment. While these mouse models are valuable tools, some have limitations due to species differences between mice and humans. In contrast to most human cells, mouse cells have a higher basal metabolic rate, shorter lifespan and active telomerase (16), all resulting in differential susceptibility to cancer between the two species. Xenograft models, in which cancer cell lines derived from patients are injected into immunodeficient mice, are often used for preclinical drug testing (17, 18). However, tumors produced by cell line xenograft models lack the heterogeneity and tumor microenvironment (TME) seen in human tumors.

Given the significant influence of the tumor microenvironment on immune cells, a better model is needed to study cancer immunology and immunotherapy. Patient-derived tumor xenograft (PDX) models preserve the genetic and cellular heterogeneity of the tumor, as well as some aspects of the tumor microenvironment (19). For this reason, PDX models are increasingly used in cancer research. Yet unfortunately, PDX models similarly lack crucial components of the human immune system such as circulating T and B cells. To study the biology and efficacy of cancer treatments, a more accurate recapitulation of both the TME and the human immune system is needed. To this end, humanized mouse models have been developed in which immunodeficient mice are reconstituted with representative subsets of human immune cells (20–22).

To study human ILCs and their role in cancer, humanized mouse models can serve as useful tools. In this review, we will discuss the role of ILCs in cancer, existing humanized mouse models that can be leveraged for more precise studies of ILCs, and therapeutics that employ ILCs against cancer along with mouse model considerations for accurate assessment of those therapies.

## Innate Lymphoid Cells

The origins of innate lymphoid cells (ILCs) in humans were elucidated from reconstitution of ILC groups in patients with hematopoietic stem cell transplantations (23) and in humanized mouse models engrafted with human CD34+ stem cells (24). Human ILCs are derived from Lin− CD34+ CD45RA+ CD117+ IL-1R1+ RORγt+ hematopoietic progenitors (25, 26) and represent a family of developmentally-related cells involved in immunity and in tissue development and homeostasis. ILC subgroups can remain circulating in the body or become tissue-resident (24). In 2013, Spits et al. proposed a classification for ILCs (27). According to this classification, these largely tissue-resident cells (28) are divided into three groups, based on similarities to adaptive T helper (Th) cell subsets. ILCs are distinguished by the reliance on transcription factors that dictate their development and maintenance and by their ability to produce signature Th cytokines mirroring those of the Th cell subsets (27). This section provides an overview of the different human and murine ILC lineages and their complex roles in cancer; following sections will elaborate on the humanized mouse models available to study ILC-based immunotherapies and drugs that influence ILC activation directly or indirectly.

### Group 1 ILCs

Group 1 ILCs are characterized by their capacity to produce IFN-γ and their dependence on transcription factor T-BET (27, 29). Group I ILCs include conventional NK cells and subsets of innate lymphocytes, termed ILC1s, which differ from NK cells in their phenotypes, locations, functions, or transcription factor dependence (30). One important difference between both human and murine NK cells and ILC1s is that NK cells express the transcription factor Eomesodermin (EOMES), which is important for their differentiation and function (27, 29, 31).

In contrast to ILC1s, which are mainly tissue-resident cells, NK cells are found in both peripheral blood and tissues (30). Human peripheral blood NK cells were originally divided into two subsets, CD56^bright^ and CD56^dim^ NK cells. More recently, additional distinct populations of tissue-resident NK cells have been described in human and murine tissues such as the gut, kidney, and liver, each displaying tissue-specific phenotypes and functions (32–35). Some NK cells in mice and humans express Fc receptors, which are functional surface molecules that bind to the conserved regions of antibodies and can induce killing of antibody-coated target cells (36).

The phenotypes and functions of ILC1s depend on the tissue microenvironment, and ILCs with a mixed NK cell and ILC1 phenotype have been observed in humans (29, 30, 37). For instance, human intra-epithelial ILC1 (ieILC1) cells possess features shared by conventional NK cells and helper ILC1; they produce IFN-γ and express both transcription factors EOMES and T-BET (30). Both NK cells and ILC1s represent a first line of defense for humans and mice against infections with viruses and certain bacteria, and are essential for the clearance of intracellular microbial infections (38–40). NK cells appear to be more potent in mediating cytotoxicity, while the primary role of ILC1s is the production of pro-inflammatory cytokines (30, 40).

### Group 2 ILCs

Human and murine ILC2s require the transcription factor GATA-binding protein 3 (GATA-3) for their differentiation and maintenance, similar to Th2 cells, and produce cytokines such as IL-5 and IL-13 in response to activation by IL-25 and IL-33 (27, 41, 42). Human ILC2s express CRTH2 and high levels of CD161, whereas most mouse ILC2s express ST2 (29). ILC2s in both mice and humans are important in the immune defense against helminth infections and in the pathogenesis of asthma (41, 43–45).

### Group 3 ILCs

Group 3 ILC3s in humans and mice produce IL-17 and IL-22, similarly to Th17 cells, and are dependent on the transcription factor retinoic acid receptor-related orphan receptor-γt (RORγt; encoded by *RORC*) for their development and function (27). Group 3 ILCs are abundant at mucosal sites and are involved in immune responses to extracellular bacteria and the containment of intestinal commensals. Human ILC3s produce IL-22, by which they promote intestinal homeostasis (46). Both human and mouse ILC3s can also produce granulocyte–macrophage colony-stimulating factor (GM-CSF) (7). Two subsets of ILC3s can be distinguished on the basis of cell surface expression of NKp44 (also known as NCR2) in humans and NKp46 (also known as NCR1) in mice, termed NCR+ or NCR- (41).

### ILCs and Cancer

As the scientific community continues to unravel the biology of ILCs in cancer, many studies have investigated the efficacy of targeting or employing ILCs as a cancer therapy. Among ILC1s, NK cells are heavily studied for their potential in cancer immunotherapy (47). However, the role of ILCs in tumor immunity is yet to be fully elucidated. ILCs are enriched in many human cancers (48, 49) and there is increasing evidence showing that they exhibit phenotypic and functional plasticity. For instance, after TGF-β exposure, both murine and human NK cells can convert to an ILC1-like phenotype (50–52). ILC lineage plasticity has also been observed; human ILC2s can convert to an ILC3 phenotype with IL-1β, IL-23, and TGF-β stimulation (53). Additionally, Gury-BenAri et al. found that upon perturbation of the mouse microbiome by administration of broad spectrum antibiotics, the transcriptional profiles of mouse intestinal ILC1s and ILC2s became more similar to that of ILC3s (54). ILC plasticity has potential consequences on tumor growth and behavior. For example, a decrease in mouse ILC1s and transdifferentiation within ILC2 subtypes correlated with colorectal cancer progression (55, 56).

Recent investigations into the complex biology of ILCs have revealed the strong influence of the tumor microenvironment (TME) on ILC responses (57). For example, human tumor cells under stressful TME conditions upregulate MICA, MICB, and ULBP1-6, which are ligands for the NK activating receptor NKG2D (58). Chronic engagement of NKG2D can result in NK cell tolerance (59). Furthermore, the TME can adopt an immunosuppressive environment by exploiting cytotoxic T lymphocyte associated protein 4 (CTLA-4), programmed cell death protein 1 (PD-1), and other immune cell reprogramming pathways (60). These pathways are further discussed in the ILC Immunotherapeutics section.

The hypoxic, immunosuppressive milieu of the TME has been shown to cause immune cell dysfunction, leading to treatment failure (61, 62). Certain cytokines within the TME can also prevent antitumor activity by ILCs. IL-33, a cytokine that activates ILC2s, has been shown to be pro-tumorigenic in humans and mice by promoting tumor growth, metastasis, and angiogenesis (63, 64). Its presence in the TME causes ILC2s to produce IL-13, which recruits myeloid-derived suppressor cells (MDSCs) to induce tumor tolerance (65, 66). Similarly, IL-23 can induce ILC3s to express immunosuppressive IL-17 and IL-22 in colon cancer models (67). It is critical to account for the role of the TME when studying the effects of ILCs and ILC treatments on cancer, and strategies to target and specifically block these inhibitory pathways may represent potential new immunotherapies.

## Humanized Mouse Models

### Mouse Models for Improved Human Immune System Engraftment

**Figure 1:**
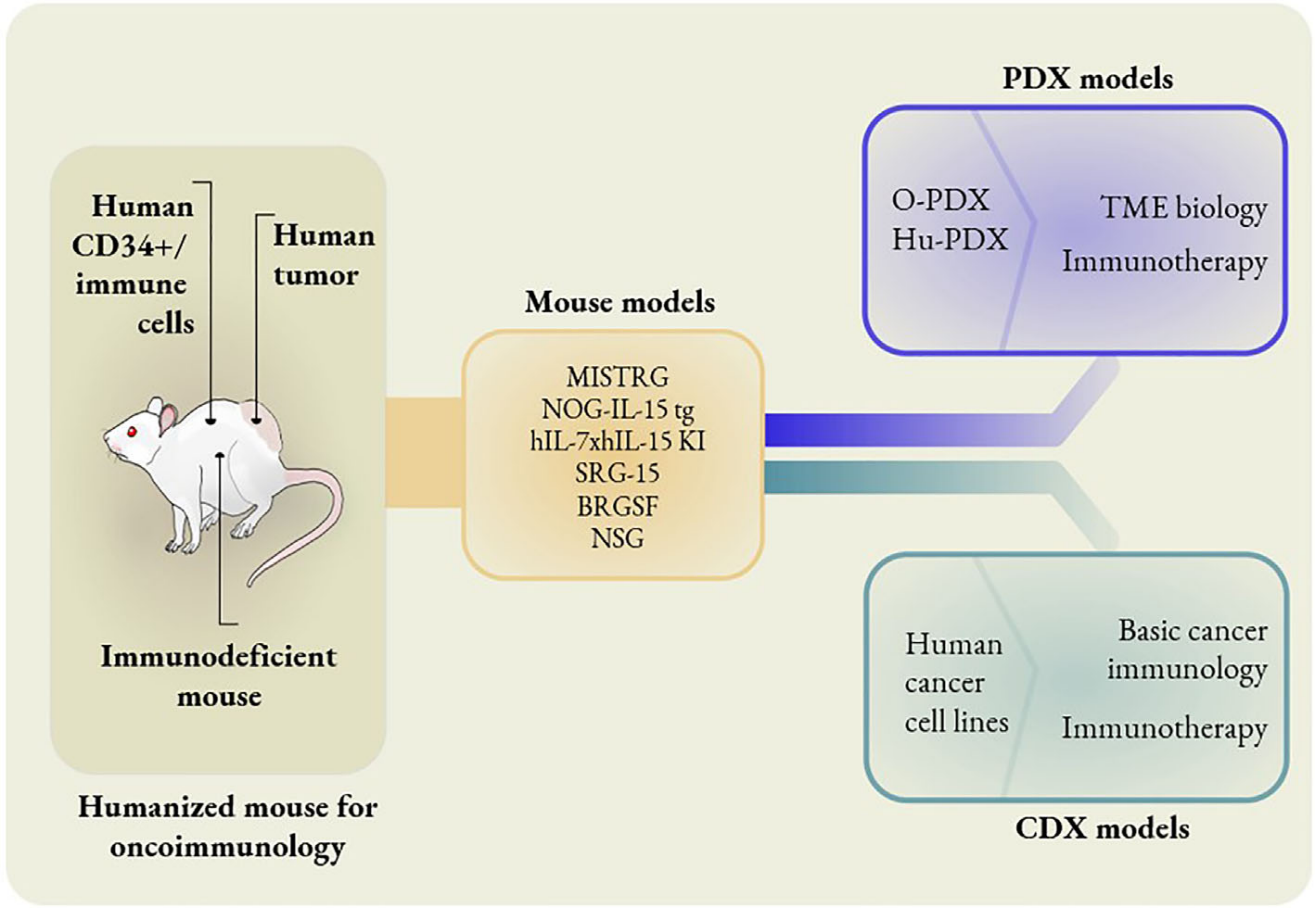
Different mouse models to study oncoimmunology. In knock-in models, mouse genes are replaced with human counterparts. MISTRG mice have human genes replaced that encode M-CSF, IL-3, GM-CSF, TPO and SIRPα. NOG-IL-15 tg mice have human IL-15 transgene expression. hIL-7xhIL-15 double knock-in mice express human IL-7 and IL-15. SRG-15 mice have the mouse IL-15 gene replaced with the human IL-15 gene. BRGSF mice are Flt3-deficient mice in a BRGS background with exogenous administration of human Flt3L. The O-PDX model consists of orthotopic patient-derived xenografts placed in MISTRG mice. The Hu-PDX mouse model consists of patient-derived xenografts placed in NSG mice with reconstituted human immune systems. Human cancer cell lines can be used in place of PDX in basic cancer immunology and immunotherapy studies. However, PDX models are ideal for the studies of TME biology and certain immunotherapies, e.g. combination immunotherapies.

Humanized mice were originally defined as mice bearing functional human genes, cells, or tissues. In more recent years, the term humanized mice has generally been used for genetically modified immunodeficient mice that are permissive to the development of human immune cells (**Figure 1** and **Table 1**). In this review, we briefly discuss immunocompetent mice carrying functional human genes, but our primary focus is on immunodeficient mice bearing human immune cells, as these are significantly more useful for cancer immunotherapy research. Early humanized mouse models were generated by transferring human peripheral blood mononuclear cells (PBMCs), hematopoietic stem and progenitor cells (HSPCs), or fetal hematopoietic tissues into the severe combined immunodeficient mouse strain, CB17-*scid* (75–77). CB17-*scid* mice are deficient in the *Prkdc* gene, which encodes the catalytic subunit of the DNA-dependent protein kinase. The *Prkdc* gene product is crucial for V(D)J recombination to generate functional T and B cells. The absence of T and B cells in *Prkdc*-deficient mice promotes host acceptance of xenografts. However, CB17-*scid* mice have NK cells and other innate immune cells that can restrict efficient engraftment of human immune cells.

**Table 1:** Humanized mice to study ILC-cancer interactions.

Mouse Model	Human Tumor Cells Administered	Human Cells Engrafted	Human Lineage Reconstitution (*lineages with improved reconstitution compared to NSG mice)	References
**MISTRG**	**Me275 melanoma cells**	**CD34+ HSPCs**	*monocytes, *macrophages, *DCs, T, B and *NK cells	(21)
**SRG-15**	**Raji tumor cells and K562 tumor cells**	**CD34+ HSPCs**	myeloid cells, *T, B *NK cells and *ILCs	(68)
**NOG-IL-15 Tg**	**NCI-N87 human gastric cancer cell**	**Peripheral blood NK cells and *in vitro*-expanded NK cells**	*NK cells	(69)
**hIL-7xhIL-15 KI**	**-**	**CD34+ HSPCs**	T and *NK cells	(70)
**BRGSF**	**-**	**CD34+ HSPCs**	*myeloid cells, T, B, DC, *NK cells and *ILCs	(71)
**O-PDX (MISTRG)**	**Neuroblastoma**	**CD34+ HSPCs**	*NK cells	(72)
**Hu-PDX** **(NSG)**	**Lung adenocarcinoma**	**CD34+ HSPCs**	**T, B and NK cells**	(73)
**HTM** **(NSG)**	**Breast cancer**	**CD34+ HSPCs**	**T, B, NK cells and macrophages**	(74)

The development of a non-obese diabetic-*scid* (NOD-*scid*) mouse model led to increased human immune cell engraftment levels, compared to CB17-*scid* mice (78). Later, it was reported that the better engraftment in NOD-*scid* vs. CB17-*scid* is due a polymorphism in the signal-regulatory protein α (*SIRPa*) gene (79, 80). The polymorphism allows for engagement of SIRPα on mouse myeloid cells to CD47 on human cells. This gives the “do not eat me” signal and inhibits phagocytosis of human cells by mouse myeloid cells. Thus, in NOD-*scid* mice, the SIRPα polymorphism supported better development of human hematopoiesis (79). However, the residual activity of NK cells and other innate cells in the NOD-*scid* mice prevented optimal engraftment of human cells. This was overcome with the usage of mice with germline mutations in the interleukin 2 receptor subunit gamma (*Il2rg*) gene, which encodes a receptor component called the common gamma chain. This mouse strain, termed NOD-*scid* Il2rg^-/-^ (NSG), is hospitable to transplanted human immune cells because the host mice completely lack NK cells (81).

### Humanized Knock-in Models to Study Cancer

Mouse strains harboring human gene knock-ins have also been developed to allow for the robust development of human immune cells in the mouse microenvironment. As previously described, the NOD-*scid* mouse strain contains a polymorphism in SIRPα, which reduces phagocytosis of engrafted human cells. With this knowledge, researchers attempted to improve the engraftment of human cells with transgenic expression of human SIRPα in the mice (82). Continued genetic manipulations of mouse strains led to an advanced mouse model called MISTRG which exhibits enhanced engraftment of human immune cells by replacing several mouse cytokines genes with corresponding human genes (21). In the MISTRG strain, mouse genes are replaced with human genes encoding M-CSF, IL-3, GM-CSF, thrombopoietin and SIRPα. These cytokines support the survival of myeloid and lymphoid cells in mouse peripheral blood and tissues.

IL-15 is an essential cytokine for the development and differentiation of NK cells. Human NK cells show poor engraftment or impaired development in mice, as mouse IL-15 is not sufficient for the development of fully functional human NK cells (83, 84). Although there is no expression of human IL-15 by mouse cells in MISTRG mice, the engrafted human monocytes and macrophages produce human IL-15 to support the endogenous development of human NK cells. Rongvaux et al. used the MISTRG mouse model for the *in vivo* investigation of NK cell activity against melanoma tumor xenografts (21).

Because of the importance of IL-15 in NK cell development and survival, routine injections of recombinant human IL-15 have also been used to promote survival of human NK cells after adoptive transfer (85). In one study, researchers differentiated NK cells *in vitro* from CD34+ HSPCs obtained from human cord blood. With subcutaneous injections of recombinant human IL-15 every 48 hours, they then demonstrated that adoptive transfer of human NK cells can control the growth of ovarian cancer xenografts in NSG mice and promote survival of the mice (85). However, recombinant human IL-15 is expensive and routine injections every 2-3 days can be laborious. Consequently, transgenic and knock-in mice expressing human IL-15 have been developed such as NOG-IL-15 Tg, hIL-7xhIL-15 KI NSG mice, and SRG-15 mice (68, 70).

The NOG-IL-15 Tg mouse model is generated from the NOD/Shi-*scid*-IL-2Rγ^null^ mouse background with the addition of human IL-15 transgene expression (69). This enables long term maintenance of transferred human NK cells isolated from peripheral blood. Using this model, Katano et al. showed that the transfer of *in vitro*-cultured NK cells in the presence of anti-Her2 antibody can suppress Her2-positive gastric cancer (69). To refine the murine host for human NK cell development *in vivo*, double gene knock-in of human IL-7 and IL-15 was done in NSG mice, which were termed hIL-7xhIL-15 KI NSG mice (70). These IL-15 producing mice are useful tools for the assessment of tumor models and combination immunotherapies involving NK cells.

A particularly attractive HIS model is called SRG-15 and involves two human gene replacements to encode human IL-15 and SIRPα. Upon injection of human CD34+ HSPCs, the SRG-15 mice can develop human T cells, B cells, NK cells and myeloid cells. Importantly, the SRG-15 mouse model allows for study of human tissue-resident immune cells, including the development of tissue-resident CD8+ T cells (IELs) and tissue-resident ILC subsets (68). The secretion of human IL-15 from mouse stromal and epithelial cells in the SRG-15 mice was sufficient to produce functional human NK cells following CD34+ HSPC engraftment (68). Furthermore, the NK cells from the reconstituted human immune system in SRG-15 mice (following CD34+ HSPC engraftment) were shown to successfully hinder CD20+ Raji tumor growth with co-administration of rituximab (68). This demonstrates that the SRG-15 mice model can be used to establish and evaluate novel combination therapy protocols.

Di Santo et al. developed another HIS mouse model to study human ILC, called Flt3-deficient BALB/c *Rag2^−/−^Il2rg^−/−^Sirpa* ^NOD^ (BRGSF). Flt3, the FMS-related tyrosine kinase 3, is a cytokine crucial for dendritic cell (DC) homeostasis. In mice lacking Flt3, the endogenous mouse DCs will no longer compete with CD34+ HSPC derived human DCs for Flt3 ligand (Flt3L) signaling. Upon exogenous administration of Flt3L, the expanded human DCs in this mouse strain provide the cytokine environment for the development of NK cells and other ILCs (71). Specifically, the researchers engrafted human CD34+ HSPCs in BRGSF mice and were able to isolate phenotypically and functionally mature human NK cells and other ILC subsets (71).

Notably, out of the many HIS mouse models described in this section, the endogenous development of human NK cells from CD34+ HSPC has only been shown in hIL-7xhIL-15 KI NSG, MISTRG, SRG-15, and BRGSF mice. This is important to consider when selecting a humanized mouse model for the study of any immunotherapy that may rely on modulation of NK cell or ILC behavior, or the survival of adoptively transferred NK cells or ILCs. In conclusion, the mouse models discussed here serve as useful tools for the study of NK cell development and cytotoxicity, and some can even be used to study the development and behavior of other ILC subsets.

### Cancer Cell Line Derived Xenograft (CDX) Models for ILC Studies

In cancer cell line derived xenograft (CDX) models, established human cancer cell lines are subcutaneously transplanted into mice to form tumors. During the injection, tumor cells can be mixed with effector cells such as NK cells to evaluate the impact of the human immune cells on tumors *in vivo*. Alternatively, instead of co-injection of effector immune cells and tumor cells, effector cells can be injected after successful engraftment of tumor cells. One such study demonstrated the effective elimination of B-cell Non-Hodgkin lymphoma tumors with anti-CD20 chimeric antigen receptor (CAR) NK cells. To establish the CDX, Raji-Luc cells, a human Non-Hodgkin lymphoma cell line expressing luciferase, were injected into NSG mice and allowed to grow for several weeks. NK cells were expanded from peripheral blood NK cells, engineered to express the anti-CD20 CAR construct, and injected into the NSG mice bearing Raji-Luc cell line xenografts. Treatment with anti-CD20 CAR NK cells showed significant reduction in tumor size, as demonstrated by the decrease in luciferase signal from the tumors. The study also demonstrated that CAR NK cells could inhibit dissemination of the tumor into other organs (86). Cany et al. established an acute myeloid leukemia (AML) xenograft model by injecting GFP and luciferase expressing K562 leukemia cells (K562.LucGFP) intrafemorally in NSG mice. They showed that NK cells, derived from umbilical cord blood, could target the leukemia cells residing in the bone marrow (87).

Cancer cell lines are advantageous for their ease in genetic manipulations and for establishing early proof of concept studies, but continuous passage of cell lines in 2D culture could result in the loss of epithelial to mesenchymal transition (EMT) potential (88). The tumors generated from cell lines also fail to reflect tumor vascularization and tumor microenvironment observed in human cancers (89). Fortunately, patient-derived xenograft models can overcome some of these drawbacks (90).

### PDX Humanized Models to Study ILCs

Patient-derived tumor xenografts are becoming an increasingly popular tool to dissect the intrinsic properties of cancer initiating cells and the interaction between tumor and immune cells *in vivo* (91). Cancers are heterogenous mixtures of cells containing malignant cells, nonmalignant stromal cells, vascular endothelial cells and immune cells. Initial cancer drug development studies did not consider the involvement of the immune system, as most of the studies were performed in immunodeficient mice with tumor explants (92). The emerging improved PDX models involve the transplantation of both tumor explants from patients and human immune cells to establish a human immune system in the mouse host.

Several studies have attempted co-transplantation of human HSPCs and tumors in mice. One such model is orthotopic patient-derived xenograft (O-PDX) for neuroblastoma, in which MISTRG mice undergo double transplantation of CD34+ HSPCs and neuroblastoma xenografts. This allows for long-term hematopoiesis and PDX engraftment simultaneously (72). The NK cells in this model exhibit antibody-dependent cell-mediated cytotoxicity (ADCC), which shows potential for preclinical testing of different monoclonal antibodies in neuroblastoma (72). In a separate study, Meraz et al. also co-transplanted cord blood derived CD34+ HSPCs and lung cancer PDX to form a NSG mouse model with a humanized immune system and PDX, which they named Hu-PDX (73). The reconstituted humanized immune system showed an antitumor response of T cells but not NK cells after administration of the checkpoint inhibitor antibody pembrolizumab (73). Thus, such a model can be used for studying immune responses to cancer and developing immunotherapy treatments against cancer.

Another category of PDX models are immunoavatar cancer models, which are highly suited for preclinical trials of immunotherapy and can also be used to develop personalized treatment strategies. In this model, human immune cells (such as PBMCs) and tumor tissue (e.g. melanoma) are taken from the same patients or individuals in autologous fashion. This model more accurately recapitulates the unique immune responses against tumors originally occurring in the patient, as they are from the same source of origin (93, 94). One issue with this model, however, is that it suffers from GVHD responses in which human T cells from the engrafted PBMCs mount immune responses against mouse cells after several weeks (92, 94).

Overall, these mouse models aid in proof-of-concept studies to better understand immune cell interactions with tumors *in vivo*. Engrafted PDX tissues demonstrate pathohistological and genetic resemblance to the original tumor, in addition to preserving parental solid tumor architecture (95). These properties make them an attractive model for personalized therapy testing, pre-clinical drug screening and basic cancer research (96).

### Limitations of Humanized Mouse Models for Cancer Research

Although humanized mouse models act as excellent models to study solid tumors, there are a few shortcomings that still need to be addressed. Many PDX transplant methods are subcutaneous, even if the original tumor was not located in that type of tissue. As a result, the environment around the tumor lacks organ-specific factors and the chronic inflammatory milieu. Developing solid tumors in the abdomen or by orthotopic transplantation into organs can also make it challenging to measure tumor size or assess tumor growth *via in vivo* imaging analysis. A potential utility of humanized cancer models is to interrogate simultaneous development of immune cells and cancer cells *in vivo*. Yet even though tumor cells and HSPCs can be co-transplanted intrahepatically into newborn humanized mice at same time, the development of the tumor sometimes precedes the reconstitution of multilineage human immune cells (74).

Mice bearing PDX could be a useful tool to dissect the role of human ILCs in cancer. However, these studies must reconcile the differences between mouse and human biology. While the original human cancer cells survive and proliferate within a PDX, over time the human stromal cells within the tumor are replaced by mouse stromal cells; this can affect the maintenance and behavior of tumor-infiltrating human immune cells (97). Hence, humanized mouse models reconstituted with multiple human immune cell lineages and engrafted with tumors are being developed to facilitate their application in designing better immunotherapy strategies.

## ILC Immunotherapeutics Studies in Murine Hosts

### Mouse Model Development and Considerations for Antibody-Based Therapies

**Figure 2:**
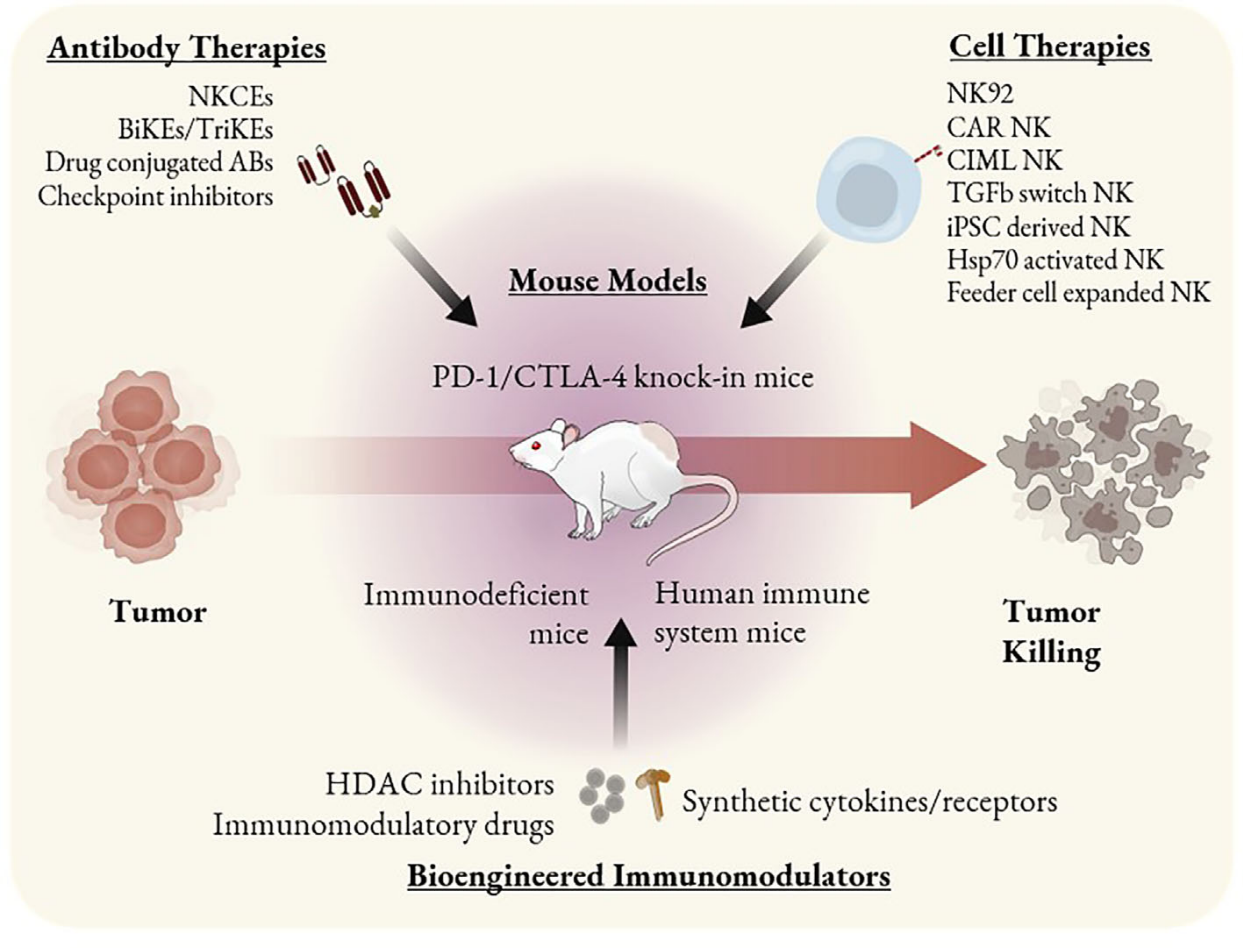
ILC based immunotherapeutics and mouse models as tools to study them. Immunotherapies that involve ILC antitumor activity can be categorized as antibody-based, cell-based, or other bioengineered immunomodulators. Mouse models such as PD-1 and CTLA-4 knock-in can be used to study checkpoint inhibitors. SCID mice bearing PDX or CDX can be used to study the efficacy of single agent cell therapies such as CAR NK cells or NK92 cells. HIS mice are the most optimal tool to study combination therapies or immunomodulators that may alter the behavior of multiple immune cell types.

Antibody-based immunotherapies are less expensive than cell-based therapies and represent a promising area to explore as combination therapeutics, since they can be administered alongside another therapeutic agent that stimulates the adaptive or innate immune system for a synergistic effect (98). A treatment that activated both ILCs and adaptive immune cells in a safe and specific manner could potentially avoid the possibility of antigen escape, be more likely to succeed in controlling metastases, and induce a systemic immune response and immune memory. However, these treatments are difficult to study in immunocompetent mice as most clinically relevant antibodies are highly specific for their human targets and thus do not exhibit cross-reactivity to their murine counterparts (99). For these reasons, researchers use humanized mice as a more accurate tool to study complex immune responses during antibody treatments. Antibodies being studied for their ability to modulate NK cell and ILC behavior include checkpoint inhibitors, Bispecific Killer cell Engagers (BiKEs), Trispecific Killer cell Engagers (TriKEs), NK Cell Engagers (NKCEs), and other novel recombinant antibodies such as drug-conjugated antibodies (**Figure 2**).

Checkpoint inhibitors block an inhibitory ligand on a tumor cell or the cognate receptor on a tumor-targeting immune cell to prevent the inhibitory signal from impacting the immune cell (100). Checkpoint molecules such as PD-1 and CTLA-4 can modulate the functionality of both adaptive and innate immune cells (101). For example, secretion of cytokines such as IL-5 and IL-13 by human ILC2s (102) or degranulation and secretion of IFN-γ by human NK cells (103) can be inhibited. NK cells express inhibitory receptors that recognize MHC class I molecules (KIRs in humans and Ly49s in mice). Human NK cells also express CD94/NKG2A, PD-1, TIM3, TIGIT, and LAG3 in certain contexts (104, 105). Other human ILCs also express inhibitory receptors in both normal and pathological conditions (106). ILC1s express TIM-3, LAG-3, CD96, and TIGIT; ILC2s express PD-1 and KLRG1; and ILC3s express TIGIT and CD96 (65). Blockade of these pathways *in vivo* can result in the reactivation of NK cells and ILCs, which may generate a strong antitumor response (105). Human inhibitory receptors such as KIRs are difficult to study in immunocompetent mouse models, since they diverge structurally between human and mouse NK cells and do not exhibit cross-reactivity - murine Ly49s would not bind to human MHC and human KIRs would not bind to murine MHC (107). As a result, early work surrounding NK/ILC checkpoint inhibitors relied on the use of immunodeficient mice with transplanted human tumor cells and single human immune cell types - a time consuming and limited approach.

A major advance towards targeting immune checkpoints was the generation of humanized CTLA-4 or PD-1 knock-in mouse models. Murine cells are modified to express the human form of the protein of interest under the same genetic regulation as its murine counterpart; the mice retain an intact functional murine immune system with the exception of the single altered protein and intracellular signaling through these molecules is largely preserved (108, 109). These models recapitulate immune modulation by tumor cells because human CTLA-4 is capable of interacting with mouse B7-1 and B7-2 (108) and human PD-1 is capable of interacting with mouse PD-L1 (110). Since human PD-1 interacts in subtly different ways with human PD-L1 compared to murine PD-L1 (111), a further improvement to the accuracy of these models is a mouse that expresses both human PD-1 and human PD-L1.

These mice are not considered human immune system (HIS) mice as they do not possess human immune cells, but they represent a robust model system that can be utilized to validate the binding and antitumor efficacy of antibodies against individual human checkpoint molecules. Mice expressing human CTLA-4 and dual humanized mice expressing human PD-1 and CTLA-4 or both human PD-L1 and PD-1 without expression of murine PD-L1 or PD-1 are now commercially available (112). Knock-in models are a useful tool for studying direct and secondary effects of targeted antibody therapies on tumor growth *in vivo*, minimizing off-target toxicities, and optimizing antibody binding kinetics to decrease the frequency of severe immunotherapy-related adverse events.

To study the effects of checkpoint inhibitors on human immune cells, immunodeficient mice are required. Marasco et al. used NSG mice with transplanted human T and NK cells to study the efficacy of CAR T cells that secrete anti-PD-L1 antibodies, so they could observe the effects of the antibodies on NK cells alongside T cells and tumor cells (113). However, NSG mice are not optimized to ensure NK cell engraftment. Models such as the MISTRG, SRG-15, and NOG-IL-15 Tg mice will further enable longer-term study of NK cell responses to antibody therapies as they can provide a constant cytokine signal to ensure NK cell survival (69). Future research using humanized mice should include the screening and optimization of a wide variety of Fc regions, receptor targets, and drug conjugates to strongly induce ILCs to kill tumor cells.

In addition to checkpoint inhibitors, other antibodies are in development that directly activate NK cells. BiKEs induce NK cell killing by binding to CD16 on the surface of NK cells and a tumor antigen simultaneously, while TriKEs target CD16, a tumor antigen, and an IL-15 molecule to activate the NK cells (114). By binding to CD16 and colocalizing tumor cells with NK cells, the antibodies trigger ADCC pathways and induce target cell killing. These antibodies show promise *in vitro* and *in vivo*, but most clinical research has been restricted to hematologic malignancies (115). To study the antibodies in mice, researchers have used xenogenic models such as NSG mice injected with human ovarian cancer cells and NK cells (116). Another novel NK cell stimulating antibody is a trifunctional NKCE that binds to NKp46 and CD16 on NK cells and a tumor antigen on target cells. Anti-CD20 NKCEs targeting NKp46 exhibited strong efficacy against solid tumors and intravenously injected Raji B cells in SCID mice (117). Drug-conjugated antibodies can also be used for immunotherapy, such as brentuximab vedotin which contains a microtubule depolymerizing agent and a variable fragment targeting CD30 on tumor cells (118). Microtubule inhibitors have cytotoxic effects on cancer cells and also induce maturation in some innate immune cells; these drug-conjugated antibodies were first tested for *in vivo* efficacy in SCID mouse xenograft models (119).

While antibody therapies have proven to be successful in clinical trials and data shows they are able to improve the activity of NK cells that enter a tumor, antibodies are unable to directly increase recruitment of immune cells to the tumor (105). Rather, they rely on activation of tumor infiltrating immune cells to generate chemotactic signals that will recruit and activate additional immune cells (120). However, this strategy is not always successful, as the immunosuppressive TME is capable of dampening the activation induced by the antibodies and preventing a strong immune response (121). Much more work needs to be done in order to improve the homing of ILCs to tumors and their activation within the TME, and mice with human immune systems are a necessary tool for these studies.

NSG or SCID mice transplanted with NK cells are sufficient to test the direct activation of NK cells by antibodies and the resulting tumor cell killing. However, they do not enable researchers to study whether these NK cells could induce the activation or recruitment of other tumor-resident or circulating immune cells through cytokine secretion or other mechanisms. This is because they do not contain diverse human immune cell subsets and therefore do not accurately represent the immune milieu present in a human tumor. For a more thorough assessment of broad immune activation after administration of these recombinant antibodies, HIS mice optimized for NK/ILC survival and proliferation would be required.

### ILC-Based Cell Therapies and Mouse Model Considerations

Adoptive cell therapies for cancer, while at first dominated by adaptive immune cells, are beginning to show promise using NK cells. Unfortunately, it is difficult to accurately model the long term influence of adoptive cell therapies on tumor progression in immunodeficient mice. Many ILCs require cytokine stimulation to survive in the absence of other immune cells, and administering those cytokines frequently can be costly and time consuming (70). For example, NK cells will survive and proliferate in NOD Rag gamma (NRG) immunodeficient mice without exogenous cytokine administration only if the mice are also transplanted with autologous human cord blood immune cells (122). Additionally, tumors grown in immunodeficient mice lack human intratumoral immune cells. Thus, it is impossible to determine whether adoptively transferred cells will activate other innate or adaptive immune cells in the TME - a critical factor for developing a systemic immune response and immune memory (34). Many cell therapies are also difficult to test in a precise manner using the murine immune system because their human cell homologs are different in ways that range from subtle to potentially significant, or artificial receptors such as CARs must be modified to function within murine signaling pathways.

The most ideal models to study the potential success or failure of adoptively transferred ILCs are humanized mice that have been optimized to ensure the survival and expansion of ILCs in the murine host, such as NOG-IL-15 Tg mice with transplanted human PBMCs or MISTRG, SRG-15, or BRGSF mice reconstituted with CD34+ stem cells. These transplant models enable long term studies of cell survival, differentiation, and proliferation without the added cost of cytokine injections or the need to engineer cells to produce their own cytokines (70). They also enable researchers to study a broad range of ILCs in parallel in the context of their therapy, as multiple populations can be induced from a single injection of fetal pluripotent stem cells rather than having to transplant them individually. For example, Ishikawa et al. used IL-6 knock-in mice engrafted with human CD34+ stem cells to study the effects of IL-6 on co-activation of macrophages and T cells (123). However, it is worth noting that mouse models endogenously expressing human cytokines can contain supra-physiological concentrations of those cytokines, which may alter the results of the studies and make results more complex to interpret (124).

ILC-based cell therapies currently in development rely on phenotypically modulated NK cells and ILCs, CAR NK cells, or NK cell lines (**Figure 2**). Phenotypic modulation of these cells is possible because NK cells and ILCs respond to cytokines, growth factors, pathogens, and other external stimuli in varied manners, and this can be exploited to enhance the efficacy of cell therapies or enable production of clinically relevant doses of cells. For example, briefly activating NK cells with IL-12, IL-15, and IL-18 can improve their long-term cytotoxicity. These cells, known as cytokine-induced memory-like (CIML) NK cells, exhibit increased proliferation, persistence, memory-like functionality, and IFN-γ production (125, 126) and show increased cytotoxicity towards many cancer cell types including ovarian cancer cells, leukemia cell lines, and primary human AML blasts (127, 128).

Cytokine-induced phenotype modulation can also be leveraged alongside growth factors to differentiate NK cells from induced pluripotent stem cells (iPSCs) (129). iPSC-derived NK cells are capable of ADCC, and CAR NK cells from this source are currently in preclinical or phase I/II testing (130). However, since there are large numbers of developmental pathways involved in these pipelines, it is difficult to perfectly replicate them using murine cells. It is equally difficult to study the interactions of human iPSC-derived NK cells with other immune cells accurately in immunodeficient mice. HIS mice enable more rigorous studies of the effects of adoptive cell therapies on other immune subsets.

Several unique subsets of NK cells are also of great interest for cancer therapies. These include NK cells, which lack expression of the intracellular signaling protein FcεRγ, called gNK cells. gNK cells appear to be induced by CMV infection (131) and exhibit increased ADCC function; they are being studied for their ability to enhance antibody therapies in B cell leukemia and lymphoma (132). Researchers are also developing methods for *ex vivo* NK cell activation to improve cell therapies. These strategies include culture with heat shock protein 70 (Hsp70), TKD peptide, and IL-2, which has been studied using both murine and human NK cells (133), and co-culture with feeder cells such as K562 that can be altered to express stimulatory molecules such as OX40 ligand (134) or IL-21 (135).

The most striking successes of ILC-based cell immunotherapies have involved CAR NK cells. CAR NK cells target surface molecules on tumor cells and exhibit a more favorable safety profile than CAR T cells (136). An anti-CD19 CAR NK cell that produces IL-15 achieved a 73% response rate in HLA mismatched patients with non-Hodgkin’s lymphoma or chronic lymphocytic leukemia. These cord blood derived CAR NK cells could show promise as a standardizable off-the-shelf therapeutic (137). Additional CAR NK cell therapies currently in clinical trials include cells targeting mesothelin, CD22, PSMA, and NKG2D ligands on tumor cells, and others currently in development include variations in cytokine production, intracellular signaling domains, and costimulatory molecules (138). CAR NK cells producing IL-15 were tested for early *in vivo* efficacy in a Raji lymphoma NSG xenograft model (138), but this model did not enable researchers to assess whether other immune cells were activated by the CAR NK cells. Another type of CAR used with NK cells is a TGF-β chimeric switch receptor which uses TGF-β to activate, rather than inhibit, NK cells in the TME (139). This receptor was studied in NSG mice with transplanted neuroblastoma cells, CAR NK cells, and intraperitoneal administration of IL-2. Future work should incorporate HIS mice to determine whether CAR NKs/ILCs can activate other immune subsets circulating in the bloodstream or residing within the TME.

NK cell lines are another area of interest for cell therapies. NK92 is an immortal cell line that was isolated from a patient with a rare NK cell lymphoma (140). CAR NK92 cells represent a potential cell therapeutic that is simple to standardize and produce in bulk. NK92s were assessed against malignant melanoma in SCID mice and showed significant potential (141). The first in human trial involving irradiated NK92 cells used anti-CD33 CAR NK92s for patients with relapsed and refractory acute myeloid leukemia, and no significant adverse events were observed (142). However, it is not yet clear whether these cells will function well against solid tumors. Due to the short lifetime of irradiated cells, it also remains to be seen whether they are capable of initiating an antitumor response that could generate long-term immunological memory. This is difficult to model in mice without humanized immune systems, since the effect of the irradiated cells on tumor size or survival might be brief or insignificant but their impact on other immune cells could be what drives tumor eradication.

Non-irradiated NK92 cells could be given to patients if they were transduced with suicide genes to ensure they could be killed if they began proliferating too rapidly (143). Researchers are studying non-irradiated Smad3-silenced NK92 cells (NK-92-S3KD) in xenograft mouse models to assess their efficacy against hepatoma and melanoma (144). Other cell lines that mimic human NK/ILC1 behavior include NK101 (145), NK3.3, YTS, and NKL (146). However, it will be difficult to assess their efficacy against solid tumors without a robust *in vivo* model that includes other immune cells due to the drastic impacts of the TME on suppression of immune cytotoxicity and the highly interactive nature of cell therapies when considering tumor-resident immune cells. Complex tumor-immune and immune-immune interactions are difficult to model using immunodeficient mice, but are critical in predicting the success or failure of cell therapies. For these reasons, HIS mice represent a more optimal tool for studying NK cell therapy *in vivo*.

## Discussion: Bioengineered Immunomodulators and Targeted Drug Delivery as New Frontiers

Modeling potential therapeutics using standard immunodeficient mice with one or two transplanted human immune cell types is not a sufficiently rigorous approach. These mice inhibit the ability of researchers to broadly identify indirect effects of their drugs on other immune cells as a result of the primary effects on their target cell of interest, and tumors grown in them from cell lines or transplanted tissue do not contain representative tumor-resident immune cell populations. For both combination and single agent therapies involving precise modulation of ILCs, HIS mice optimized to support the engraftment and development of multiple immune cell types represent a significant improvement for modeling possible responses from tumor cells and immune cells. Here we discuss the next generation of ILC-related immunotherapies and the mouse models required to study them.

Several new immunotherapies in development could enhance ILC antitumor functionality (**Figure 2**) but require humanized mice for accurate modeling of their effects. Histone deacetylases (HDACs) and HDAC inhibitors are able to alter transcription of genes related to tumor growth or suppression (147) and induce cell cycle arrest in cancer cells (148). HDACs have been shown to enhance ADCC of NK cells against tumor cells (149), but some HDAC inhibitors can diminish NK cell longevity and cytotoxicity (150) while others enhance NK cell functions (151). These treatments are difficult to study accurately since they impact both tumor cells and tumor-resident immune cells in different ways—not all of which are replicated by murine cells. Further studies in mice optimized for NK cell survival will enable determination of the effects of HDACs on tumor and NK cells simultaneously.

Synthetic cytokines can also enhance NK/ILC immunotherapies, since the broad mechanisms of action of endogenous cytokines limit the doses that can be safely administered (152). Artificial cytokines can be engineered to exhibit modified binding profiles, enhanced stability and specificity, increased activation of immune cells, multifunctional capacity, and lower induction of autoimmunity (153). For example, synthetic IL-2 and IL-2R pairs enable specific activation of only the engineered immune cells in a patient (154). Synthetic cytokines can also be targeted to tumor cells to improve activation of immune cells only within the TME (155). Artificially tethered membrane-bound IL-15 can improve longevity and cytotoxicity of NK cells (156), and membrane-bound IL-21 on feeder cells can modulate NK cell phenotype when expanding them for autologous therapy (135). In the future, synthetic cytokine and receptor pairs could be used to specifically activate CAR NK cells or ILCs only when they are adjacent to tumor cells. Since a major advantage of bioengineered cytokines is their ability to activate multiple types of immune cells simultaneously and synergistically, HIS mice represent an optimal tool for studying their effects on NK cells and ILCs in addition to other immune cells.

Non-cytokine immunomodulatory drugs (IMiDs) ranging from snake venom (157) to antimicrobial peptides (AMPs) such as cathelicidin (158) have been tested for their ability to improve NK cell antitumor functions. Drugs such as lenalidomide and pomalidomide can enhance antitumor ILC behavior as well (159, 160). Bryostatins are protein kinase C modulators that are currently being engineered to improve CAR NK functionality (161). These and many other drugs have the potential to broadly or specifically activate NK cells or ILCs in addition to other immune cells within the TME. However, only small numbers of murine studies have been carried out thus far to determine their ability to activate tumor-resident ILCs and influence tumor eradication *in vivo*. To accurately assess them, HIS mice optimized for NK/ILC proliferation such as NOG-IL-15 Tg mice transplanted with human PBMCs should be used.

Finally, almost all of the therapies discussed in this review can be further enhanced through targeted drug delivery. This enables researchers to consider thousands of drugs for cancer therapy that were previously considered too toxic to administer systemically. These drugs can be targeted to cancer cells or activated only within the TME using delivery mechanisms such as oncolytic viruses (162), coated nanoparticles (163), and ultrasound activation (164). This also increases the quantity of drugs to which the tumor can be exposed, potentially increasing their efficacy. Oncolytic viruses can be used as checkpoint inhibitor delivery vehicles, limiting autoimmunity and other disorders that might originate from systemic delivery (165). NK/ILC therapies can also be strengthened by using transplanted cells as a delivery vehicle or enhancing their function through separately targeted drugs. This could improve their killing capacity, localization to tumors, or activation within the TME. Researchers have used NK92 cells to bring drug-loaded nanoparticles to a solid tumor and block inhibitory signals in the TME (166), and local delivery of chemoattractants such as CCL20 or CXCL16 can increase tumor infiltration by ILCs (167). There is a great deal of future work that should be done in the drug delivery space to enhance ILC-related therapies, and HIS mice are the most ideal tool for early stage optimization and assessment because these mice can facilitate the survival and proliferation of ILC/NK cell therapies and their tumors will contain diverse immune cell populations.

## Concluding Remarks

In order to solve critical unanswered questions surrounding the dynamic interactions between ILCs, solid tumors, and other tumor-resident immune cells, researchers should leverage humanized mouse models to increase the accuracy and utility of their studies. New generations of mice have been optimized to ensure ILC survival and enable development of multiple immune cell types simultaneously, both of which are critical for accurately studying the direct effects of immunotherapies as well as their indirect effects on other immune cells. Topics related to ILC-cancer biology that are in need of further investigation include: determining why current immunotherapies show limited efficacy in solid tumors, identifying methods to subvert the signals in the TME that are suppressing ILCs, assessing the unknown roles and plasticity of ILCs in cancer both endogenously and after adoptive transfer, and understanding the signals communicated between ILCs and other immune or tumor cells and how those correlate with tumor growth or eradication. For all of these endeavors, humanized mice represent a useful tool to study these complex questions.

The broad range of biotechnology that is being invented at breakneck speed is inspiring but also daunting. It seems virtually impossible to test all of these potential immunotherapy-enhancing agents in an accurate manner - especially in combination with each other - using standard immunodeficient mouse models. The potential utilities of combination therapies are much broader than those of single agent therapies, so it is important to study them accurately. While the primary effects of these therapies can be assessed on single types of immune or cancer cells by transplanting them into immunodeficient mice, that approach misses a large portion of information that is critical to predicting their efficacy against solid or liquid tumors: the secondary effects these cells will have on other cells in the TME or bloodstream. Improved *in vivo* modeling using humanized mice would drastically increase our ability to determine which of these technologies should be translated to a clinical setting for human trials and potentially save the lives of many patients, along with the costly sums of money required to test individual drugs. HIS mice expressing molecules such as IL-15 that improve ILC survival and proliferation will further enable researchers to design and optimize cellular and molecular immunotherapies.

## Author Contributions

NH, IM, and JS conceived and organized the outline of the review. NH, IM, UM-N, IK, QT, and JS contributed to the writing. IM created the figures. All authors contributed to the article and approved the submitted version.

## Funding

This work was supported by grants from the National Institutes of Health (R35DE030054; R01DE027750), which is funding the JBS laboratory for research on cancer immunotherapy.

## Conflict of Interest

JS is the scientific co-founder and member of the scientific advisory board of Indapta Therapeutics; however the science presented here is not related to the focus of the company. UM-N is the founder of Conference Fund; however, the science presented here is not related to the focus of the company.

The remaining authors declare that the research was conducted in the absence of any commercial or financial relationships that could be construed as a potential conflict of interest.
